# Mental distress among Liberian medical staff working at the China Ebola Treatment Unit: a cross sectional study

**DOI:** 10.1186/s12955-015-0341-2

**Published:** 2015-09-26

**Authors:** Li Li, Changli Wan, Ru Ding, Yi Liu, Jue Chen, Zonggui Wu, Chun Liang, Zhiqing He, Chengzhong Li

**Affiliations:** Department of Nursing, Eastern Hepatobiliary Surgery Hospital, Second Military Medical University, No. 225, Changhai Road, Yangpu District, Shanghai, 200438 People’s Republic of China; Department of Orthopaedic Surgery, Shanghai Changzheng Hospital, Second Military Medical University, Shanghai, People’s Republic of China; Department of Anesthesiology, Shanghai Changhai Hospital, Second Military Medical University, No. 168, Changhai Road, Yangpu District, Shanghai, 200433 People’s Republic of China; Outpatient Department, Shanghai Changhai Hospital, Second Military Medical University, No. 168, Changhai Road, Yangpu District, Shanghai, 200433 People’s Republic of China; Department of Cardiology, Shanghai Changzheng Hospital, Second Military Medical University, No. 415, Fengyang Road, Huangpu District, Shanghai, 200003 People’s Republic of China; Department of Infectious Diseases, Shanghai Changhai Hospital, Second Military Medical University, No. 168, Changhai Road, Yangpu District, Shanghai, 200433 People’s Republic of China; Second patch of Medical Team of the Chinese People’s Liberation Army to Liberia, Beijing, People’s Republic of China

## Abstract

**Background:**

Ebola virus outbreak in West Africa not only triggered a grave public health crisis, but also exerted and induced huge mental distress on medical staff, which would negatively influence epidemic control and social rebuilt furthermore. We chose the local medical staff working at the China Ebola Treatment Unit (ETU) to explore the severity of potential mental distress and involved potential causes.

**Methods:**

A descriptive study using the Symptom Check List 90 - Revised (SCL90-R) questionnaire to assess psychological health status was conducted among 52 Liberian medical staff. Global indices, including Global Severity Index (GSI), Positive Symptom Total (PST) and Positive Symptom Distress Index (PSDI), and nine subscales based on 90 inquiry items were compared among gender, work duty and other subgroups. Data were analyzed using Graphpad Prism and SPSS software.

**Results:**

Mental distress among participants was not very serious; only PSDI, paranoid ideation and interpersonal sensitivity numerically increased relative to changes in other categories. While male medics and those responsible for cleaning and disinfection showed significant increases in scores for psychological dimensions, such as obsessive-compulsive, anxiety, phobic anxiety, interpersonal sensitivity, paranoid ideation and positive symptom total.

**Conclusions:**

Data of this study implies that the psychological health status of medical staff within the special social environment of an Ebola treatment unit should warrant more attention.

## Background

Beginning in March 2014, the Ebola virus outbreak in West Africa triggered a grave public health crisis that hit Guinea, Liberia and Sierra Leone particularly hard. Case count doubled every 4 weeks [1] as a reflection of slow global response and outbreak underestimation [2].

Although the crisis has already passed, what could we learn from it and are we ready for the further public crisis? In addition to well-known undermined readiness of local public health systems caused by long-term poverty, human rights abuses and civil, fear and misinformation about Ebola significantly hampered epidemic control efforts and accelerated viral spread. According to data from several studies, the last contributing factor is often overlooked and common not only in affected regions but in far-away countries such as the United Kingdom and the United States [3, 4]. As documented early in Ebola outbreaks in tropical African countries, medical staff shortages are exacerbated by the mental distress that medics experience as targets of fear by the affected community [5, 6].

Because severity of mental distress among local medical staff has been inadequately defined previously in similar crisis, the present cross-sectional study was done to evaluate Liberian medical peers under such circumstance, which will provide the first-hand data and help to prepare furthermore regional or global public crisis. These Liberian medical peers worked in China Ebola Treatment Unit (ETU) that were swiftly organized and deployed by the Chinese government in an international collaboration effort to aid West Africa combat Ebola [7]. Located at Samuel Kanyon Doe Sports Stadium in Monrovia, Liberia, the ETU counts with a team that has been in place and in full operation since setup in October 2014 and could be used as a representative sample of local medical staff fighting against Ebola.

## Methods

### Study design and participants

Liberian medics working at the China Ebola Treatment Unit (SKD stadium, Monrovia, Liberia) were enrolled from March 1 to 10, 2015 if aged >18 years, willing to complete the study questionnaire, and without mental disorders. Other inclusion criterion for the participants was the ability to understand, speak, and write English, which could approve the completion of mental distress evaluation without difficulty. Since such study was done during the prevailing period of Ebola, it was very dangerous and not possible for us to go to other ETUs in Liberia to enroll more participants, the snow sampling strategy was used and we enrolled all the Liberian medics in our ETUs only according with the above inclusion criteria. All study participants provided written informed consent, and the study protocol was approved by the Biomedical Ethics Committee in the second batch of the Medical Team of the Chinese People’s Liberation Army to Liberia.

### Participant evaluation

Demographics and psychological health status data, including age, gender, education level, marital status, and whether any family member had died of Ebola within 6 months were collected from study participants. The SCL-90-R questionnaire was used as in a previous report [8], and two trained experts in the ETU mainly responsible for psychological support and fluent spoken English separately evaluated the participants’ psychological health status for about 20–25 min. English was chosen to do all the evaluation, which was widely used among the participants during the routine work in the ETU and could avoid potential information loss. Results of the 90-item SCL90-R questionnaire were classified according to 9 psychological dimensions: 1) somatization (SOM); 2) obsessive-compulsive (O-C); 3) interpersonal sensitivity (I-S); 4) depression (DEP); 5) anxiety (ANX); 6) anger-hostility (HOS); 7) phobic anxiety (PHOB); 8) paranoid ideation (PAR); and 9) psychoticism (PSY); and according to 3 global scales: the Global Severity Index (GSI), Positive Symptom Total (PST) and Positive Symptom Distress Index (PSDI). While the answers to each question included “not at all” (0) to “a little bit” (1),”moderately” (2),”quite a bit” (3) and “extremely” (4), the corresponding severity of psychological discomfort was graded as normal, mild, moderate, severe, or very severe, respectively.

Other possible independent variables considered in the analysis were working positions, occupation type and sex. And other factors were also collected such as marital status, education, religion, age and whether lost one of family member or not. Working positions included observation, treatment and cleaning. Occupations were divided into nurses and hygienists. Marital status was classified as “Unmarried”, or “Married”, and religious belief as “Christian” or “others”. Lost one of family member was defined as “Yes” or “No” following the question: “Have you lose one of your family member during the outbreak of Ebola?” Education level was classified as elementary school, middle school, high school, or college graduate and the latter two were regarded as receiving higher education. Age was classified into < 32 and ≥32-years-old.

### Definitions and scores

The validity and reliability of the SCL90-R test has been confirmed previously in studies around the world [9–14]. Overall psychological health status was evaluated using the GSI score, a global scale; average level of distress was determined as the mean scores for PST and PSDI and compared among group categories. The former PST is a count of all the items with non-zero responses, and PSDI is calculated by dividing the sum of the values of items with non-zero responses by PST [15].

### Statistical analysis

All data are expressed as mean ± standard derivation. Group means were compared by one-way or two-way analysis of variance (ANOVA) followed by a Student-Newman-Keuls test. Analysis of all data was performed using GraphPad Prism 4 and SPSS for Windows 7.0; *p* < 0.05 was considered statistically significant. In order to elucidate the potential influence of factors such as work duty, gender and job type on the psychological health status among such participants, subgroup analysis was done later.

## Results

### Demographics

Demographic characteristics of the 52 study participants are shown in Table 1. Mean age was 32.3 ± 6.7 (range, 24–50) years and 24 (46.2 %) were male, respectively; 65.4 % had higher education. Average time working at the ETU was 5.5 ± 2.0 (range, 3–10) months.

**Table 1:** Demographic characteristics of the participants (*n* = 52)

Demographic items	Status	Frequency	%
Gender	Male	24	46.2
	Female	28	53.8
Ages (years)	<32	29	55.8
	≥32	23	44.2
Working positions	Observation	19	36.5
	Treatment	22	42.3
	Cleaning	11	21.2
Job	Nurse	16	30.8
	Hygienist	36	69.2
Marriage status	Married	18	34.6
	Unmarried	34	65.4
Religious belief	Christian	34	65.4
	Others	18	34.6
Lost one of family member	Yes	11	21.2
	No	41	78.8

### Psychological dimensions among participants overall

Among participants overall, mean GSI, PST and PSDI was 0.42 ± 0.42, 24.15 ± 18.27, and 1.31 ± 0.51, respectively, and among the 9 dimension subscale categories mean scores were: SOM, 0.21 ± 0.32; O-C, 0.34 ± 0.48; I-S, 0.59 ± 0.62; HOS, 0.24 ± 0.45; PAR, 0.61 ± 0.65; DEP, 0.48 ± 0.46; ANX, 0.27 ± 0.42; PHOB, 0.59 ± 0.69; and PSY, 0.41 ± 0.48. Among the latter, PSDI, PAR and I-C showed numerical increases relative to changes in other categories (Table 2).

**Table 2:** Psychological dimensions of SCL90-R among the participants (*n* = 52)

Clinical diagnosis	Mean Score	Standard Deviation	95 % CI	
			Lower	Upper
GSI	0.42	0.42	0.31	0.53
PST	24.15	18.27	19.19	29.12
PSDI	1.31	0.51	1.17	1.45
SOM	0.21	0.51	0.13	0.30
O-C	0.34	0.48	0.21	0.47
I-S	0.59	0.62	0.42	0.76
DEP	0.48	0.46	0.36	0.60
ANX	0.27	0.42	0.16	0.39
HOS	0.24	0.45	0.12	0.36
PHOB	0.59	0.69	0.40	0.78
PAR	0.61	0.65	0.43	0.79
PSY	0.41	0.48	0.28	0.54

CI: Confidence interval, GSI: Global Severity Index, PST: Positive Symptom Total, PSDI: Positive Symptom Distress Index, SOM: somatization, O-C: obsessive-compulsive, I-S: interpersonal sensitivity, DEP: depression, ANX: anxiety, HOS: anger-hostility, PHOB: phobic anxiety, PAR: paranoid ideation, PSY: psychoticism

### Subgroup analysis of psychological dimensions

Because of heterogeneity among participants, analysis was also done among subgroups such as by work duty, gender and job type.

**Fig. 1:**
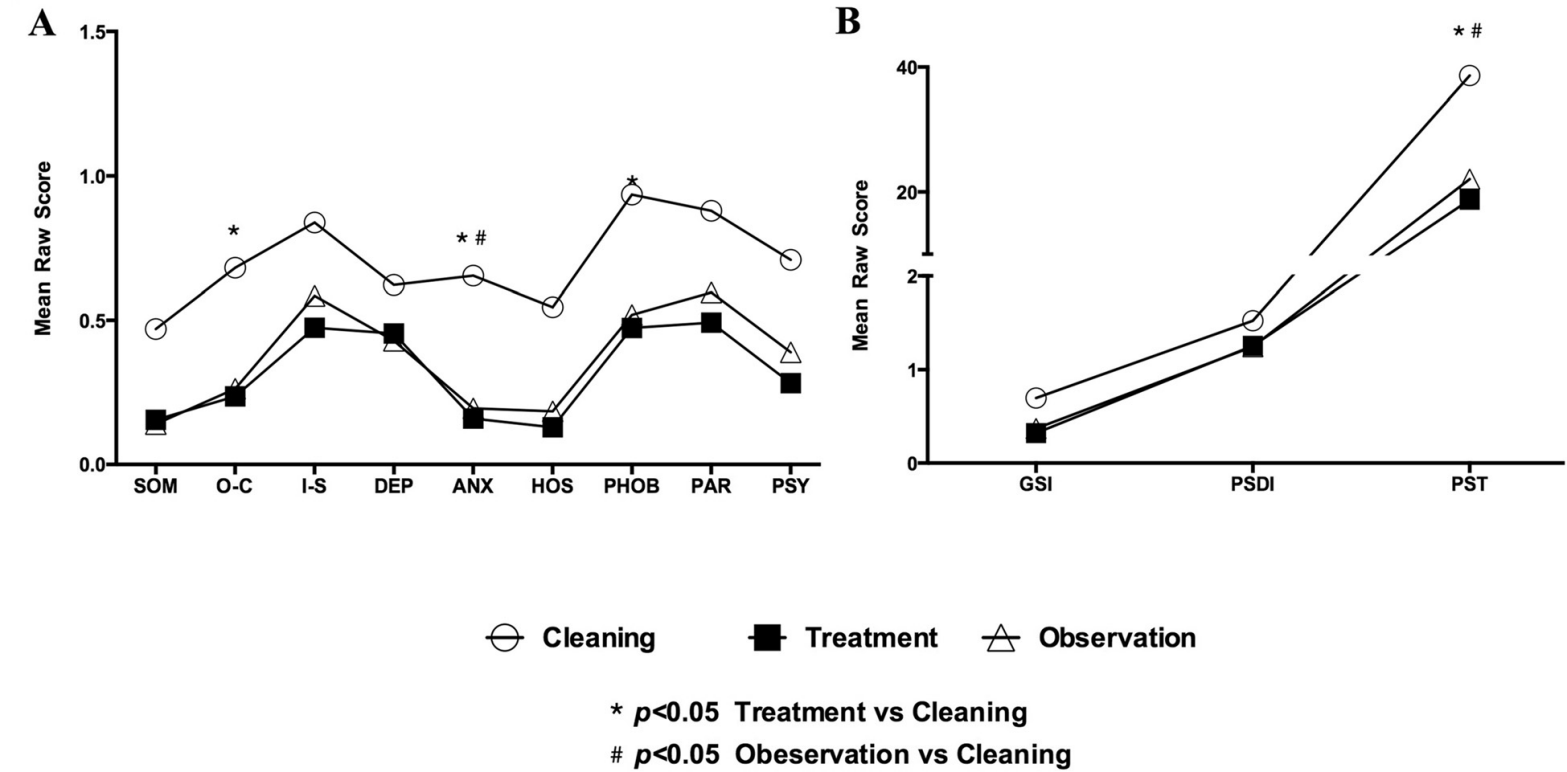
Dimensions of SCL90-R among 3 subgroups of participants working in observation and treatment ward or responsible for cleaning and disinfection. Concerning different working position, participants responsible for cleaning and disinfection showed significantly higher levels of obsessive-compulsive, anxiety, phobic anxiety (**a**) and Positive Symptom Total scales (**b**) than those in treatment ward (*p* < 0.05), while similar higher level of anxiety than those in observation ward (*p* < 0.05)

Among the work duty subgroups, local medical staff responsible for cleaning and disinfection showed significantly higher levels of obsessive-compulsive (1.68 ± 1.03 versus 1.24 ± 0.78, *p* < 0.05), anxiety (1.65 ± 0.99 versus 1.16 ± 0.52, *p* < 0.05), phobic anxiety (1.94 ± 1.19 versus 1.47 ± 1.05, *p* < 0.05) and PST categories (38.64 ± 16.61 versus 18.73 ± 17.89, *p* < 0.05) than treatment ward staff, and of anxiety level (1.68 ± 1.03 versus 1.19 ± 0.61, *p* < 0.05) and PST (38.64 ± 16.61 versus 22.05 ± 15.83, *p* < 0.05) than observation ward staff (Fig. 1a, b).

**Fig. 2:**
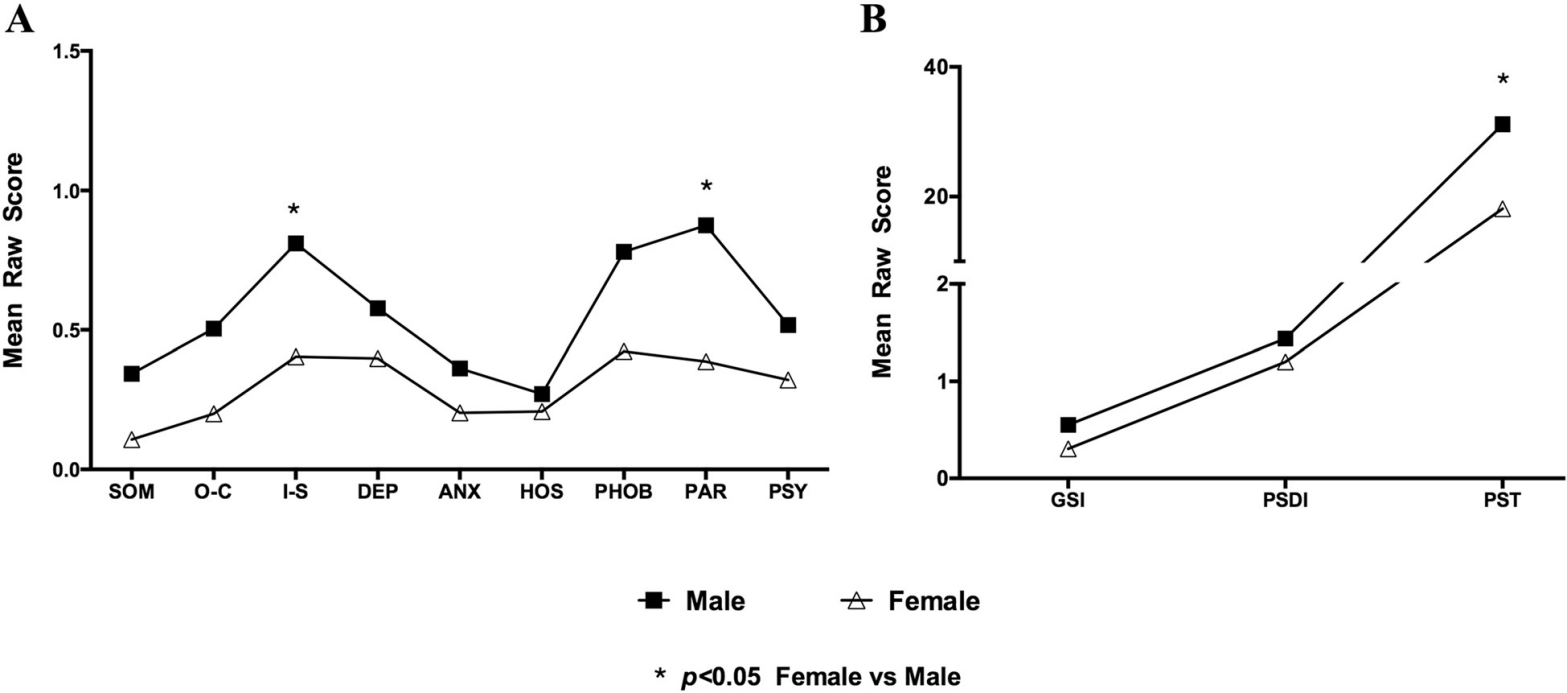
Dimensions of SCL90-R between male and female participants. More severity of interpersonal sensitivity, paranoid ideation (**a**) and Positive Symptom Total (**b**) was shown in male participants compared with female ones (*p* < 0.05)

In terms of gender differences, male participants showed significantly more severe interpersonal sensitivity (0.81 ± 0.70 versus 0.40 ± 0.48, *p* < 0.05), paranoid ideation (0.88 ± 0.75 versus 0.39 ± 0.47, *p* < 0.05) and PST (31.17 ± 19.44 versus 18.14 ± 15.08, *p* < 0.05) as compared to female ones (Fig. 2a, b).

**Fig. 3:**
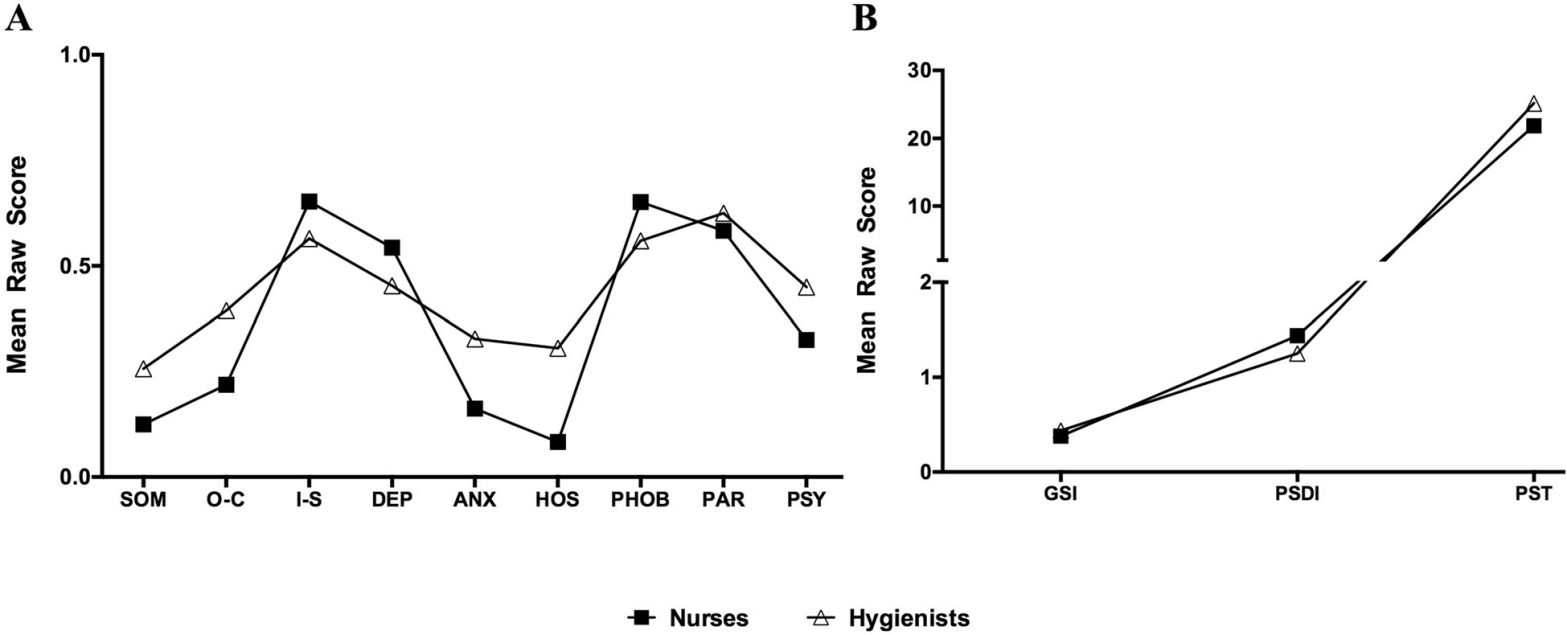
Dimensions of SCL90-R between nurses and hygienists. No difference of all the analyzed psychological dimensions was found between nurses and hygienists among neither psychological dimensions (**a**) nor positive symptom total (**b**) (*p* > 0.05)

No significant differences were apparent between nurses and hygienists for all analyzed psychological dimensions (*p* > 0.05) (Fig. 3a, b).

## Discussion

With significant support from the international community, the fight against the recent Ebola outbreak in West Africa primarily focused on promptly bringing the underestimated epidemic under control. Despite said efforts, the Ebola epidemic reached crisis levels with toll mounting by April 2015 according to WHO [16] to 25,178 cumulative case counts and 10,445 case deaths.

Prominent among the reasons cited for the Ebola virus spread across West Africa and into other continents was insufficient medical staff not only stemming from impoverished health systems in affected African countries but also fueled by severe mental stress during the epidemic [17]. Previous Ebola outbreaks in Africa had attested to the great sacrifices endured by medical staff [18–21]. The frontline staff facing infected patients has been not only at great peril of becoming themselves infected because of inadequate availability of protective gear, but also subjected to intense stigmatization by family, coworkers, and the community [22]. Effective intervention unfortunately remains elusive despite recognition of these problems and formulation of pertinent recommendations by the World Health Organization and Médecins Sans Frontières et al. [17]. The present cross-sectional study therefore was conducted among local medical staff in an attempt to gain further insight into this unmet medical need.

Data from the present study showed that mental distress among local medical staff in an ETU was not very serious. Possible reasons include: 1. At assessment time, local medical staff had worked at the ETU from 3 to 10 months, and some had even worked at an Island Clinic Centre for a while before being employed by our ETU, which made them be very familiar with Ebola virus and decreased fear about it; 2. the study was implemented from March 1 to 10, when vigilance already had been implemented without any newly lab-confirmed Ebola cases in Liberia after March 1 [16], which might significantly relieve their distress; and 3. close cooperation and early psychosocial support by our medical team and other organizations also might have contributed to mitigation of emotional distress consequences among Liberian medics. Nonetheless, subgroup analyses revealed that scores for psychological dimensions such as obsessive-compulsive, anxiety, phobic anxiety, interpersonal sensitivity, paranoid ideation and Positive Symptom Total significantly increased among male medical staff and those responsible for cleaning and disinfection. At our ETU, the cleaning and disinfection section was responsible for disinfection of recycled protective gear, garbage collection out of isolated wards, and incineration. Although personnel was not directly in contact with suspected or confirmed Ebola patients as were those at observation and treatment wards, the garbage handled was hazardous and high risk even under the protection of personal protective equipment (PPE). Furthermore, heat and smoke during incineration was not without hazards related to heat stress and dehydration, as evinced by fainting occurrences during work.

As for the male medical staff, abnormal levels of interpersonal sensitivity, paranoid ideation and PST relative to females might be derived from relative differences in workload which was physically more demanding for males, who were mainly in charge of garbage collection and cleaning within the wards. The possibility of greater negative influence by communal stigmatization against male medical staff could not be excluded.

No significant difference was apparent in mental distress despite the difference in education level between nurses and hygienists (100 % vs. nearly 50 % had higher education, respectively) possibly because: 1. Both nurses and hygienists had been intensively trained for 2 weeks at our ETU on donning, doffing, use and disposal of PPE and dealing with infected patients according to WHO recommendations, which might have changed their attitudes toward Ebola overall; and 2. As shown by studies of intensive care units [23], the abundant information exchange during dinners and night duties among staff might have fostered prompt identification and correction of potential flaws, and possibly effectively alleviated tense attitudes towards working in isolated wards with many infected patients.

Attention should be paid to increased levels of obsessive-compulsive, anxiety, phobic anxiety, interpersonal sensitivity, and paranoid ideation among local medical staff seen in the present study because of the very prevailing social stigma in Ebola affected areas. Stigmatization against medical staff working against Ebola had already fueled viral spread and hampered efficient infection control during the alert management phase not only in Africa but also in other affected countries [24–27]. To offset such stigma, Ebola infection survivors were encouraged to engage in the fight against Ebola as burial team members, contact tracers, and community educators, which benefitted affected communities [26, 28, 29]. However, despite several plans proposed by WHO and its partners aimed at resolving the problem [30], stigma against local medical staff has not received adequate attention, which might further undermine the already fragile public health system infrastructure against further possible infectious outbreaks in Africa.

The present study was limited by its observational design; the lack of a suitable reference for SCL-90R psychological dimension norms for the Liberian population; and the small study sample size which might have precluded finding significant associations among variables. Further research with normal controls and larger sample sizes is warranted.

## Conclusions

Findings of this study implied that within the specific social environment of the fight against Ebola, the psychological health status of the medical staff warrants more attention and intervention particularly among male medical staff responsible for cleaning and disinfection. As brave fighters against Ebola, local medical staffs are susceptible to being infected and they deserve and should be relieved from unnecessary mental distress stemming from social stigmatization and work related stress, both physical and mental.

## Abbreviations

ETU: Ebola Treatment Unit
SCL90-R: Symptom Check List 90 – Revised
GSI: Global Severity Index
PST: Positive Symptom Total
PSDI: Positive Symptom Distress Index
SOM: Somatization
O-C: Obsessive-compulsive
I-S: Interpersonal sensitivity
DEP: Depression
ANX: Anxiety
HOS: Anger-hostility
PHOB: Phobic anxiety
PAR: Paranoid ideation
PSY: Psychoticism (PSY)
ANOVA: Analysis of variance
PPE: Personal protective equipment
